# The IFN-γ-IDO1-kynureine pathway-induced autophagy in cervical cancer cell promotes phagocytosis of macrophage

**DOI:** 10.7150/ijbs.51241

**Published:** 2021-01-01

**Authors:** Shao-Liang Yang, Hai-Xia Tan, Tian-Tian Niu, Yu-Kai Liu, Chun-Jie Gu, Da-Jin Li, Ming-Qing Li, Hai-Yan Wang

**Affiliations:** 1 Department of Gynecology of Integrated Traditional Chinese and Western Medicine, Hospital of Obstetrics and Gynecology, Fudan University, Shanghai, 200011, People's Republic of China; 2 Department of Obstetrics and Gynecology, Zhangye People's Hospital of HeXi College, Zhangye, Gansu, 734000, China; 3 Laboratory for Reproductive Immunology, Hospital of Obstetrics and Gynecology, Fudan University, Shanghai, 200011, People's Republic of China; 4 Key Laboratory of Reproduction Regulation of NPFPC, SIPPR, IRD, Fudan University, Shanghai 200032, People's Republic of China; 5 Shanghai Key Laboratory of Female Reproductive Endocrine Related Diseases, Shanghai, 200011, People's Republic of China

**Keywords:** IFN-γ, IDO1, kynurenine, autophagy, cervical cancer, phagocytosis, macrophage

## Abstract

**Background:** Cervical cancer is a common malignant disease in female patients accompanied by activation of autophagy in tumor cells. However, the exact regulatory factors of autophagy and its effects on the immune response remain unknown.

**Methods:** The induction of autophagy in HeLa and SiHa cells treated with IFN-γ, tryptophan depletion, kynurenine and epacadostat was detected by western blot analysis and by an autophagy detection kit. Following co-culture with pre-treated HeLa and SiHa cells, U937 cells were analyzed by flow cytometry to detect CD80, CD86, CD163 and CD206 expression and the induction of phagocytosis.

**Results:** IFN-γ caused a significant increase in the autophagy levels of HeLa and SiHa cells by promoting indoleamine-2,3-dioxygenase-1 (IDO1) expression. The induction of phagocytosis in HeLa and SiHa cells and the expression levels of CD80 and CD86 in U937 cells were increased significantly following treatment with recombinant human IFN-γ. This effect was associated with the induction of tumor cell autophagy. IFN-γ treatment and IDO1 overexpression promoted tryptophan depletion and kynurenine accumulation in cervical cancer cells. The latter was more potent in inducing autophagy of cervical cancer cells and promoting phagocytosis of macrophages. *In vivo*, IDO1 overexpression restricted tumor growth in C57 mice and enhanced the induction of phagocytosis in macrophages.

**Conclusions:** IFN-γ promoted induction of autophagy and macrophage phagocytosis in cervical cancer cells possibly via IDO1 expression and kynurenine metabolism.

## Introduction

Cervical cancer is a major cause of female morbidity and mortality and is currently the second most common malignant disease in women worldwide 1, 2. The increased routine screening of the cervix has enabled the diagnosis of a high number of early stage cervical cancer patients, leading to improved disease prognosis^3^. However, when the disease progresses to the advanced stages, which includes recurrence and metastasis, the effect of surgery or radiotherapy is considerably limited. It was reported that a high amount of immune cells which infiltrate the tumor tissue and the immune microenvironment could determine the clinical outcome of cervical cancer patient 4. Therefore, immunotherapy, which has achieved significant progress in several malignant diseases, such as melanoma and non-small cell lung cancer 5, 6, is now regarded as a potential treatment strategy to improve the survival of these patients. In order to examine the potential of immunotherapy in cervical cancer, the investigation of the interaction between tumor cells and immunocytes is necessary.

Indoleamine-2,3-dioxygenase (IDO1) is an enzyme, which can convert tryptophan into kynurenine. Due to its potential to induce immune suppression of T cells in certain diseases, the application of IDO1 has been investigated extensively in clinical trials of melanoma, metastatic breast cancer and acute myeloid leukemia 7. It has been shown that IDO1 is highly expressed in cervical tumor cells 8. The assessment of the role of IDO1 in cervical cancer growth progression can provide evidence for the application of IDO1 inhibitors in clinical trials.

As an important professional antigen-presenting cell, macrophages play indispensable roles in T cell stimulation and immune regulation. Activated macrophages can enhance the antitumor effects of cytotoxic T cells, while tolerant macrophages can promote tumor growth, angiogenesis, and metastasis 9-11. Phagocytosis of macrophages is the first step in tumor specific antigen processing. The antigens that combine with the co-stimulatory molecules CD80/CD86 effectively activate CD4^+^ and CD8^+^ T cells with the exception of the major histocompatibility complex molecules. During the tumorigenesis of the cervix, human papillomavirus (HPV) can inhibit the phagocytosis of macrophages to induce immune evasion 12. A certain signal is produced by CD47, which can inhibit the phagocytosis of macrophages and can be used as an efficient target in the treatment of cervical cancer 13.

Autophagy is a highly conserved process in eukaryotic cells used to dispose unnecessary or dysfunctional components for reuse. As tumor cells are characterized by rapid proliferation, nutrition deficiency and hypoxia, their autophagy levels are usually upregulated. In certain studies, autophagy in cancer cells was reported to contribute to the immune suppression and tumor growth 14, while it has also been shown that it can promote antigen presentation and immune activation 15. In a previous study, our group demonstrated that autophagy suppression in endometrial stromal cells could inhibit the cytotoxicity of NK cells 16. However, currently there is no consensus on the role of autophagy in the progression of cervical cancer. In addition, it remains unknown whether autophagy of cervical cancer contributes to the crosstalk between cervical cancer and NK cells.

In the present study, the role of the IFN-γ-IDO1-kynurenine pathway was investigated in the induction of autophagy of cervical cancer cells and in the regulation of macrophage function. The results may provide evidence for potential treatment strategies against cervical cancer.

## Materials and Methods

### Cell culture

The HeLa, SiHa (human cervical cancer cell line), TC-1(HPV-16 E6/E7 and c-Ha-Ras co-transformed mouse lung epithelial cell line) and U937 (human monocyte cell line) were bought from American Type Culture Collection. HeLa, SiHa and TC-1 cells were cultured with DMEM/F12 and U937 in RPMI 1640 (HyClone Laboratories, Logan, UT, USA), containing 10% fetal bovine serum (Gibco Cell Culture, Carlsbad, CA, USA) and 1% Antibiotic-Antimycotic (Gibco Cell Culture, Carlsbad, CA, USA). Cells were passaged depending on their densities. The temperature of the incubator was stabilized at 37°C and CO_2_ concentration was 5%.

### Co-culture of cervical cancer cells with U937 cells

To identify whether IFN-γ, rapamycin or kynurenine treated cancer cells impact the phagocytic activity and polarization of human monocyte / macrophage cell line U937, HeLa and SiHa cells were pretreated with recombinant human IFN-γ protein (rhIFN-γ, 10ng/ml, PeproTech), rapamycin (2μmol/l, Sigma), or kynurenine (500μmol/l, Sigma) for 48 hours, supernatant was discarded and cells were washed with PBS. Then, fresh medium and U937 cells were added to the plate and cervical cancer cells were co-culture directly with U937 cells for 48 hours at the ratio of 1:1. After 48 hours, cells were harvested and analyzed by FCM.

### Western blot

Cells were washed with PBS for three times, and lysed with lysis buffer (Beyotime Biotechnology, Shanghai, China), containing protease inhibitor cocktail (MedChemExpress, Shanghai, China) and phosphatase inhibitor cocktail (MedChemExpress). Protein concentrations were detected using a BCA protein assay kit (Beyotime Biotechnology). After that, protein was diluted with loading buffer (Beyotime Biotechnology) and heated to 95°C for 10 minutes; denatured protein was stored at -20°C. For western blot, equal amounts of protein calculated according to the concentration were electrophoresed on 10% sodium dodecyl sulfate polyacrylamide gels (Epizyme Scientific, Shanghai, China), transferred to nitrocellulose membranes (BioRad, Hercules, CA, USA), blocked by 5% non-fat milk for 2 h at room temperature, and incubated with corresponding primary LC3B, IDO1 and GAPDH antibody (1:1000, Cell Signaling Technology, Danvers, MA, USA) overnight at 4 °C. The membrane was washed three times with Tris Buffered Saline with Tween 20 (TBST) for 15min and incubated with HRP-linked Anti-rabbit IgG (Cell Signaling Technology, Danvers, MA, USA) for 1 h at room temperature. After washing for three times with TBST, protein bands were wetted with Immobilon Western Chemiluminescent HRP Substrate (Millipore, Darmstadt, Germany) and detected by Luminescent Image Analyzer LAS 4000 (FUJIFILM, Japan).

### Flow cytometry (FCM)

HeLa, SiHa or U937 cells collected from wells were centrifuged at 1500 rpm for 6 min, and incubated with APC-conjugated anti-human CD45, PE-conjugated anti-human CD86, and PE/CY7-conjugated anti-human CD163, FITC-conjugated anti-human CD80, and BV421-conjugated anti-human CD206 (eBioscience, San Diego, CA, USA). Specifically, HeLa and SiHa cells were fixed, permeabilized, and then stained with APC-conjugated anti-human IDO1 antibody. After that, the cells were washed twice with PBS, and resuspended for FCM analysis. In parallel, the isotopic IgG antibodies were used as controls.

In animal experiment, tumor tissue was mechanical cut, digested with collagenase, and filtrated by sieve to prepare monoplast suspension. Cells were centrifuged at 1500 rpm for 6 min, and incubated with Percp-conjugated anti-mouse CD45, APC-conjugated anti-mouse F4/80, BV605-conjugated anti-mouse CD11b, FITC-conjugated anti-mouse CD80, PE-conjugated anti-mouse CD86, Pecy7-conjugated anti-mouse CD206 (Biolegend, San Diego, CA, USA).

Data were collected in FACS Calibur flow cytometer (Beckman Coulter CyAn ADP or Beckman Coulter Cytoflex, North Carolina, USA) and analyzed with FlowJo 7.6. Each experiment was performed for three times independently. Statistical analysis was performed by using isotype matched controls as references. Typically, less than 1% positive cells were permitted beyond the statistical marker in the appropriate controls.

### DAP Green autophagy detection

Cells were seeded in an appropriate dish overnight. Discard the supernatant and wash the cells with culture medium once. Add the diluted DAP Green solution (0.1μmol/l, Dojindo Laboratories, Japan), incubate at 37℃ for 30 minutes. Discard the supernatant and wash the cells with culture medium twice. Then add the medium in different group and treat cells for 4 hours. Discard the supernatant, dye the nucleus with DAPI (Sigma-Aldrich, USA) for 10 minutes and wash the cells with culture medium twice. Observe fluorescence and take pictures under a fluorescence microscope (Leica, Munich, Germany). Multiple fields of view were randomly selected through fluorescence microscope observation, and then the number of autophagosomes was calculated.

### Phagocytosis assays

HeLa and SiHa cells were planted in 24-well plates and treated with rhIFN-γ(10ng/ml, PeproTech), epacadostat(50nmol/L, MedChemExpress), kynurenine(500μmol/l, Sigma) or tryptophan free medium as shown in results for 48 hours. Then cells were harvested and re-suspended in PBS supplemented with 5 μmol/l of 5, 6-carboxyfluorescein diacetate succinimidyl ester (CFSE, eBioscience, San Diego, CA, USA) for 8 min at 37°C with 5% CO_2_. After washed with PBS, the CFSE-labelled cells were co-cultured with U937 cells for 2 hours at the ratio of 1:1, then cells were harvested and incubated with APC-conjugated CD45 antibody to label U937. The phagocytic ratio was tested by flow cytometry. CFSE^+^CD45^+^ cells were regarded as U937 cells which had swallowed CFSE^+^ cancer cells.

### Lentivirus transfected in HeLa and SiHa cells

HeLa and SiHa cells were transfected with IDO1 overexpression lentivirus or negative control lentivirus respectively. Briefly, HeLa and SiHa cells were seeded at a density of 5 × 10^5^ cells/well in 6-well plates and adhered overnight. At the density of 50%, cells were transfected in triplicate with lentivirus at the MOI of 1, 10, and 100. Choose appropriate MOI based on the fluorescence intensity. Puromycin was continuously used to filter the successfully transfected cells for 2 weeks until the purity was more than 90%. TC-1 cells were transfected with mouse IDO1 lentivirus in the same way.

### High-performance liquid chromatography (HPLC)-tandem mass spectrometry (LC-MS/MS) quantification of Tryptophan and kynurenine

Qualitative assessment of tryptophan metabolites by MS (metabolomics) was performed by the Institute of Biomedical Sciences, Fudan University. HeLa and SiHa cells treated with IFN-γ or transfected with IDO1 overexpression lentivirus were washed with PBS, trypsinized and collected in 1.5ml centrifuge tube. Cells were lysed by adding 300μl deionized water, freezed and thawed for three times. Add 900μl methanol to the lysis solution, well mixed and then centrifuged at 20000g for 10 minutes. Collected the supernatant and volatilized to get dry power. Samples and tryptophan/kynurenine standards were detected in TSQ-Vantage triple quadrupole mass spectrometer (Thermo), using a ShimazuLC (LC-20AB pump) system and a C18 column (250mm×2.1mm I.D., 3μm particle size, ULTIMATE). Selected reaction monitoring (SRM) scan mode was applied, and transitions evaluated were Trp, 205.09-188.0, Kyn, 209.063-94.2. The results were analyzed in Analyst Software.

### Animal mode and treatment

Animals used in this study were approved by the Ethical Committee of the Obstetrics and Gynecology Hospital, Fudan University. Four-week-old female C57BL/6 mice were obtained from Animal Laboratory (Shanghai, China). After one week's adaptation, 16 mice were randomly divided into two groups, injected subcutaneously on the back with 200μl TC-1 cells (5×10^6 cell per mouse, IDO1 overexpression or negative lentivirus transfected). When palpable, tumors were measured every other day and tumor volume was calculated as 1/2(length*width*width). Mice were euthanized via cervical dislocation. when the tumor width was over 20mm or ulceration of tumor was observed. The tumor tissue was minced, digested and subjected for FCM.

In epacadostat experiment, 28 mice were randomly divided into four groups, injected subcutaneously on the back with 200μl TC-1 cells (5×10^6 cell per mouse, IFN-γ overexpression+PBS, IFN-γ overexpression+epacadostat, negative lentivirus+PBS, negative lentivirus+epacadostat). In IFN-γ and kynurenine group, 26 mice were randomly divided into three groups, injected subcutaneously on the back with 200μl TC-1 cells (5×10^6 cell per mouse). Mice in different groups were intraperitoneal injected with epacadostat (50mg/kg), IFN-γ (100ng/animal), or kynurenine (100mg/kg) every day after the tumor was palpable. Tumor volume was detected every three days.

### Statistical analysis

All of the data are shown as mean ± SEM. Comparison between controls and treatments was analyzed by Student's *t*-test. Comparison between groups more than two was analyzed by One-way ANOVA. All analyses were performed using Graphpad Prism software (Graphpad Software, San Diego, CA, USA) for Windows. Differences were considered to be significant at *P*<0.05.

## Results

### IFN-γ induces autophagy of cervical cancer cells and promotes phagocytosis and activation of macrophages

**Figure 1:**
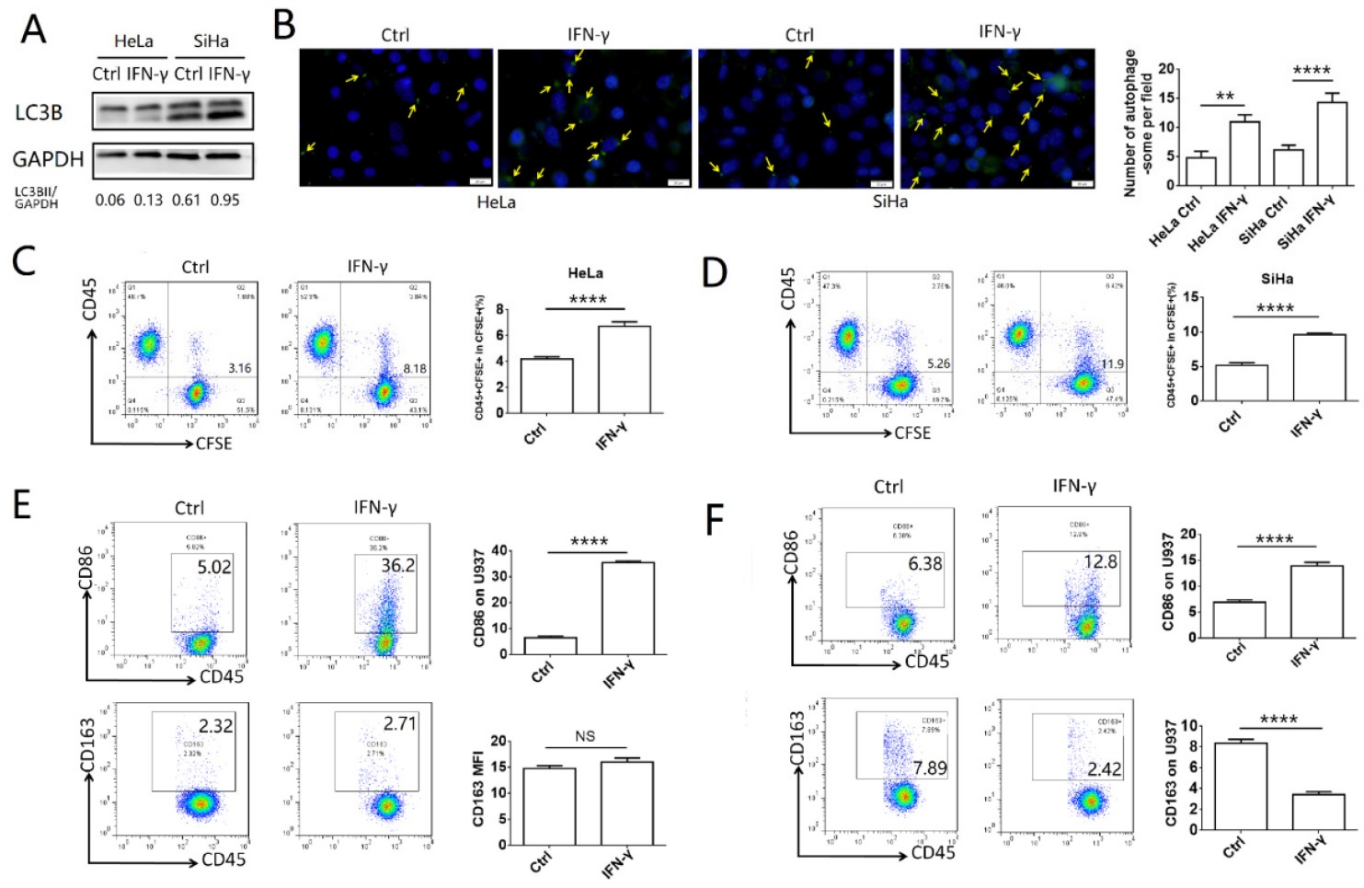
** IFN-γ induces autophagy of cervical cancer cells and promotes phagocytosis and activation of macrophage.** (A) HeLa and SiHa cells were treated with recombinant human IFN-γ at a concentration of 10ng/mL for 48 hours. LC3B was measured by western blot. (B) The autophagy level of HeLa and SiHa cells after treatment with IFN-γ was detected by DAP autophagy kit and observed with fluorescence microscope. Yellow arrows represented autophagosomes. Multiple fields of view were randomly selected through fluorescence microscope observation, and then the number of autophagosomes was calculated. (C, D) HeLa and SiHa cells were pretreated with IFN-γ or not, and then labelled with CFSE, and further co-cultured with U937 cells for 2 hours. The phagocytosis of U937 cells to HeLa and SiHa cells was tested by FCM. (E, F) HeLa and SiHa cells were pretreated with IFN-γ or not, and then co-cultured with U937 cells for 48 hours, and the expression of CD86 and CD163 on U937 cells were measured by FCM. IFN-γ: recombinant human IFN-γ. The results were expressed as mean ± SEM. NS: not significant difference, ** means *P<*0.01, and **** means *P<*0.0001.

To explore the effects of IFN-γ on the autophagy levels of cervical cancer cells and the function of macrophages, HeLa and SiHa cells were treated with 10 ng/ml IFN-γ, and the autophagic activity was assessed by western blotting and with a specific autophagosome detection kit. LC3BII was the most commonly used marker to indicate the activity of autophagy. In the present study, the expression of LC3BII was increased significantly following IFN-γ treatment in HeLa and SiHa cells, as demonstrated by the LC3BII/GAPDH ratio (**Fig. 1A**). The number of autophagosomes (green dot pointed by yellow arrow), which were detected by the green fluorescence, was also increased in the IFN-γ treated cells (**Fig. 1B**). To investigate the effects of IFN-γ on macrophages, cervical cancer cells were stained with CFSE and co-cultured with U937 cells for either 2 h to assess the levels of phagocytosis, or for 48 h to assess the polarization levels. It was found that cervical cancer cells treated with IFN-γ were more likely to be engulfed by U937 cells, while the percentage of CD45^+^CFSE^+^ cells was increased in the IFN-γ treated group (**Fig. 1C-D**). In addition, IFN-γ-treated HeLa and SiHa cells promoted the expression of CD86 in U937 cells. In pretreated SiHa cells, CD163 expression was inhibited, whereas this was not noted in HeLa cells (**Fig. 1E-F**). The expression levels of CD80 and CD206 were not significantly altered in HeLa and SiHa cells (data not shown).

### The induction of autophagy in cervical cancer cells promotes phagocytosis and the activation of macrophages

**Figure 2:**
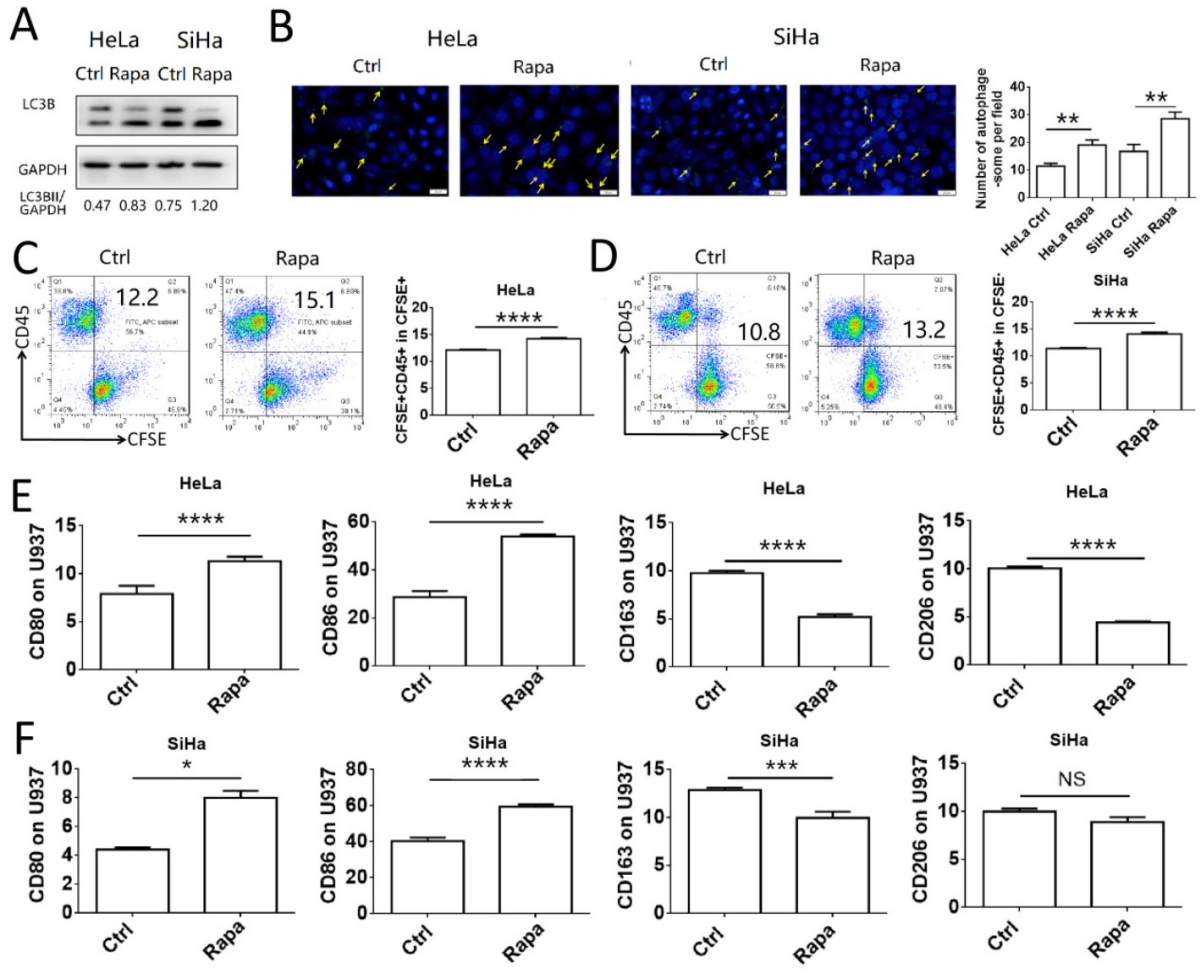
** Autophagy of cervical cancer cells promotes the phagocytosis and activation of macrophage.** (A) HeLa and SiHa cells were treated with rapamycin at a concentration of 2μmol/l for 48 hours. LC3B was measured by western blot. (B) The autophagy level of HeLa and SiHa cells after treatment with rapamycin was detected by DAP autophagy kit and observed with fluorescence microscope. Yellow arrows represented autophagosomes. Multiple fields of view were randomly selected through fluorescence microscope observation, and then the number of autophagosomes was calculated. (C, D) HeLa and SiHa cells were pretreated with rapamycin or not, and then labelled with CFSE, and further co-cultured with U937 cells for 2 hours. The phagocytosis of U937 cells to HeLa and SiHa cells was tested by FCM (E, F) HeLa and SiHa cells were pretreated with rapamycin or not, and then co-cultured with U937 cells for 48 hours, and the expression of CD80, CD86, CD163 and CD206 on U937 cells were measured by FCM. The results were expressed as mean ± SEM. Rapa represents for rapamycin, NS: not significant difference, * means *P<*0.05, ** means *P*<0.01, *** means *P<*0.001, and **** means *P<*0.0001.

To investigate the association between induction of autophagy in cervical cancer cells and macrophage function, HeLa and SiHa cells were pretreated with rapamycin and co-cultured with U937 cells. Initially, the autophagy-inducing efficiency of rapamycin was assessed in HeLa and SiHa cells. The results indicated that rapamycin increased significantly the autophagy levels in HeLa and SiHa cells (**Fig. 2A-B**). HeLa and SiHa cells treated with or without rapamycin were collected, labeled with CFSE and subsequently co-cultured with U937 cells for 2 h to assess the levels of phagocytosis. The number of cells phagocytosed by U937 macrophages was increased in the rapamycin-treated group (**Fig. 2C-D**). Following 48 h of co-culture, the polarization of U937 cells was measured by FCM. The expression levels of CD80 and CD86 in U937 cells co-cultured with rapamycin-pretreated cancer cells were increased compared to those of the control group, whereas, CD163 expression was decreased, in both HeLa and SiHa cell co-cultures (**Fig. 2E-F**). Rapamycin-pretreated HeLa cells exhibited decreased expression levels of CD206 in U937 cells, while no difference was noted in U937 cells co-cultured with SiHa control cells and SiHa cells pretreated with rapamycin. These results demonstrated that autophagy of cervical cancer cells promoted the activation of macrophages *in vitro*.

### IFN-γ induces autophagy of cervical cancer cells by upregulating IDO1 expression

**Figure 3:**
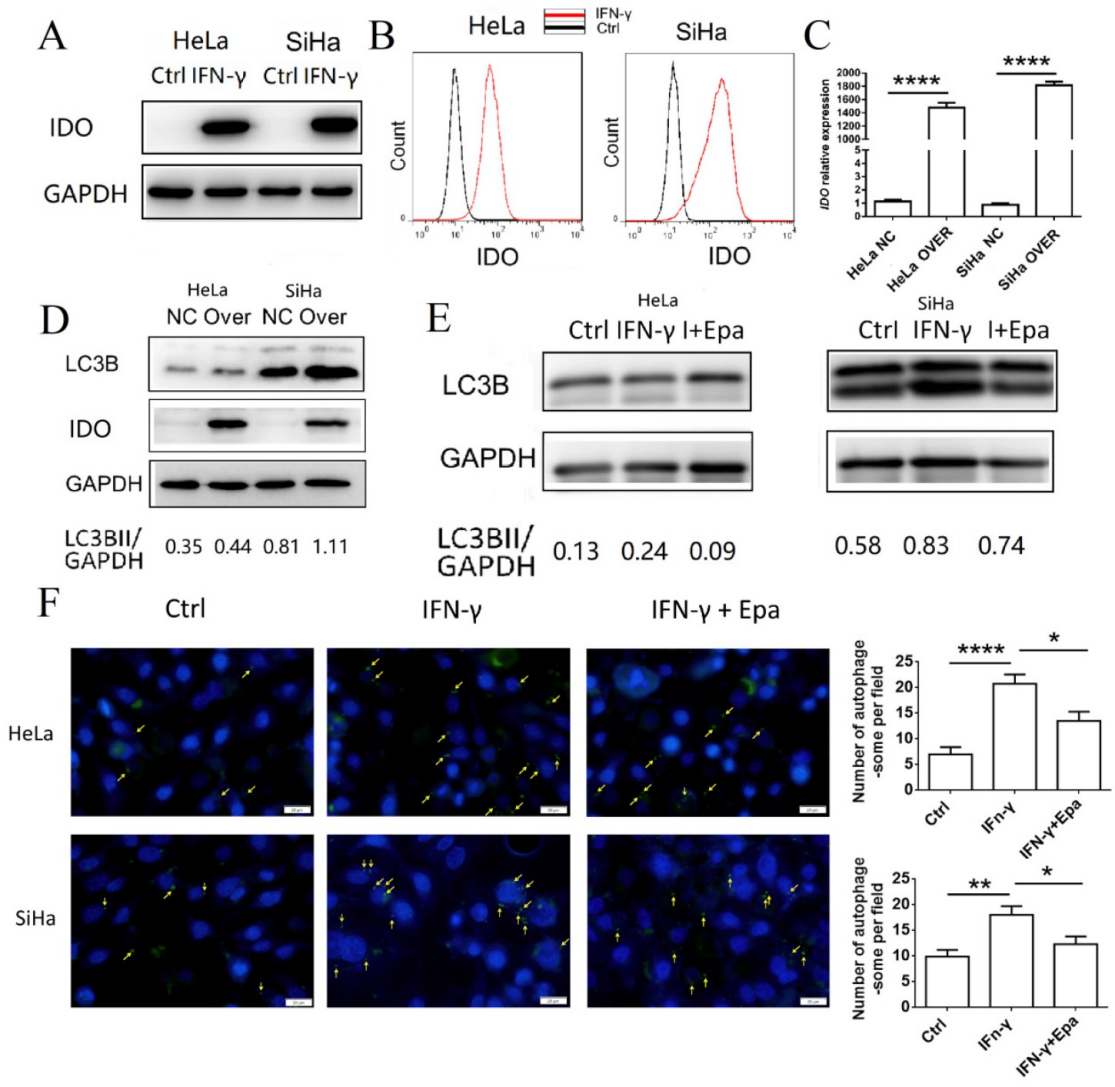
**IFN-γ induces autophagy of cervical cancer cells by up-regulating IDO1 expression.** (A, B) HeLa and SiHa cells were treated with IFN-γ or not, and the IDO1 expression was detected by western blot and FCM. (C) Transfection efficiency of IDO1 overexpression lentivirus was verified by PCR. (D) LC3B expression in HeLa and SiHa cells transfected with lentivirus was measured by western blot. (E) HeLa and SiHa cells were treated with IFN-γ at the presence of epacadostat or not, and the LC3B expression was measured by western blot. (F) HeLa and SiHa cells were treated with IFN-γ at the presence of epacadostat or not, and the autophagy level were detected by DAP autophagy kit. Autophagosomes were observed with fluorescence microscope. Yellow arrows represented autophagosomes. Multiple fields of view were randomly selected through fluorescence microscope observation, and then the number of autophagosomes was calculated. The results were expressed as mean ± SEM. Epa represents for epacadostat, I+Epa represents for IFN-γ+ epacadostat. * means *P<*0.05, ** means *P*<0.01, and **** means *P<*0.0001.

To identify the possible regulatory mechanism of IFN-γ and IDO1 on autophagy of cervical cancer cells, IDO1 expression in HeLa and SiHa cells treated with IFN-γ was detected by western blot analysis and FCM (**Fig. 3A-B**). HeLa and SiHa cells rarely expressed IDO1 without the stimulation of IFN-γ. Following stimulation, IDO1 expression was increased significantly. The identification of IDO1 overexpression was confirmed by PCR and western blot analysis (**Fig. 3C-D**). IDO1 overexpression caused a significant increase in the LC3BII expression levels of HeLa and SiHa cells (**Fig. 3D**), suggesting that IDO1 promoted the induction of autophagy of cervical cancer cells. Epacadostat, an inhibitor of IDO1, caused a significant decrease in the IFN-γ-induced cell autophagy as determined by western blot analysis and autophagy probe detection (**Fig. 3E-F**). These data indicated that IFN-γ induced autophagy of cervical cancer cells possibly by promoting IDO1 expression.

### IDO1 promotes phagocytosis of cervical cancer cells and activation of macrophages

**Figure 4:**
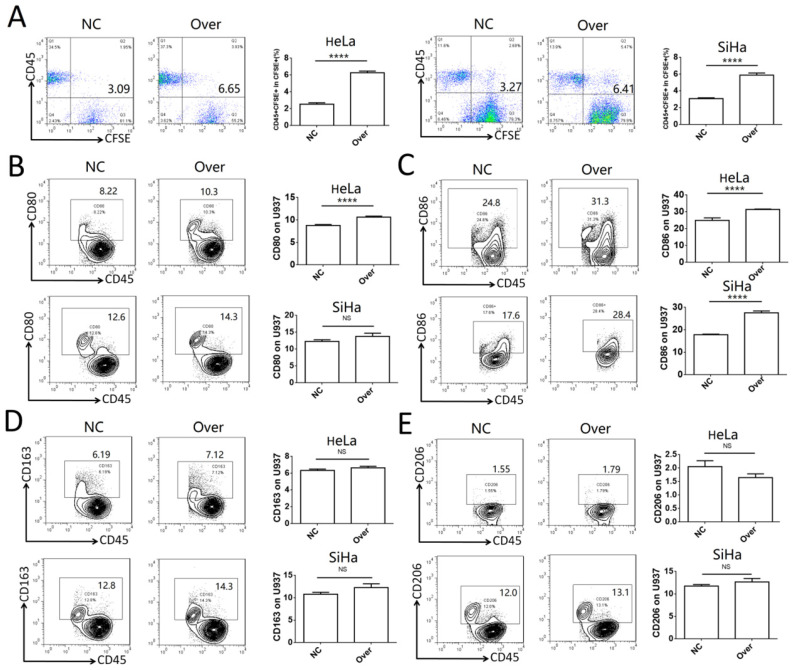
**IDO1 in cervical cancer cells promotes phagocytosis and activation of macrophage.** (A) HeLa and SiHa cells were transfected with IDO1 overexpression or negative control lentivirus, then labeled with CFSE, and further co-cultured with U937 cells for 2 hours, and the phagocytosis of U937 cells was detected by FCM. (B-E) HeLa and SiHa cells transfected with IDO1 overexpression lentivirus or negative control were co-cultured with U937 cells for 48 hours, and the CD80, CD86, CD163 and CD206 expression of U937 were detected by FCM. The results were expressed as mean ± SEM. NS: not significant difference, **** means *P<*0.0001.

To investigate the role of IDO1 in cervical cancer, the phagocytotic ability of U937 cells on control HeLa and SiHa cells or HeLa and SiHa cells overexpressing IDO1 was analyzed. Cervical cancer cells transfected with IDO1 overexpression lentivirus exhibited higher levels of phagocytosis compared to those of the control group (**Fig. 4A**). CD45 was used to distinguish cancer cells and U937 cells in the co-culture system. It was found that CD80 expression was increased significantly in U937 cells in the IDO1 overexpression group, whereas CD86 expression was increased in U937 cells co-cultured with IDO1-overexpressing HeLa cells (**Fig. 4B-C**). However, IDO1 overexpression in HeLa and SiHa cells had no effect on CD163 and CD206 expression in U937 cells (**Fig. 4D-E**). These findings implied that IDO1 promoted the phagocytosis of cervical cancer cells by macrophages, which required their activation.

### The accumulation of kynurenine and the consumption of tryptophan, which are catalyzed by IDO1, promote phagocytosis and activation of macrophages

**Figure 5:**
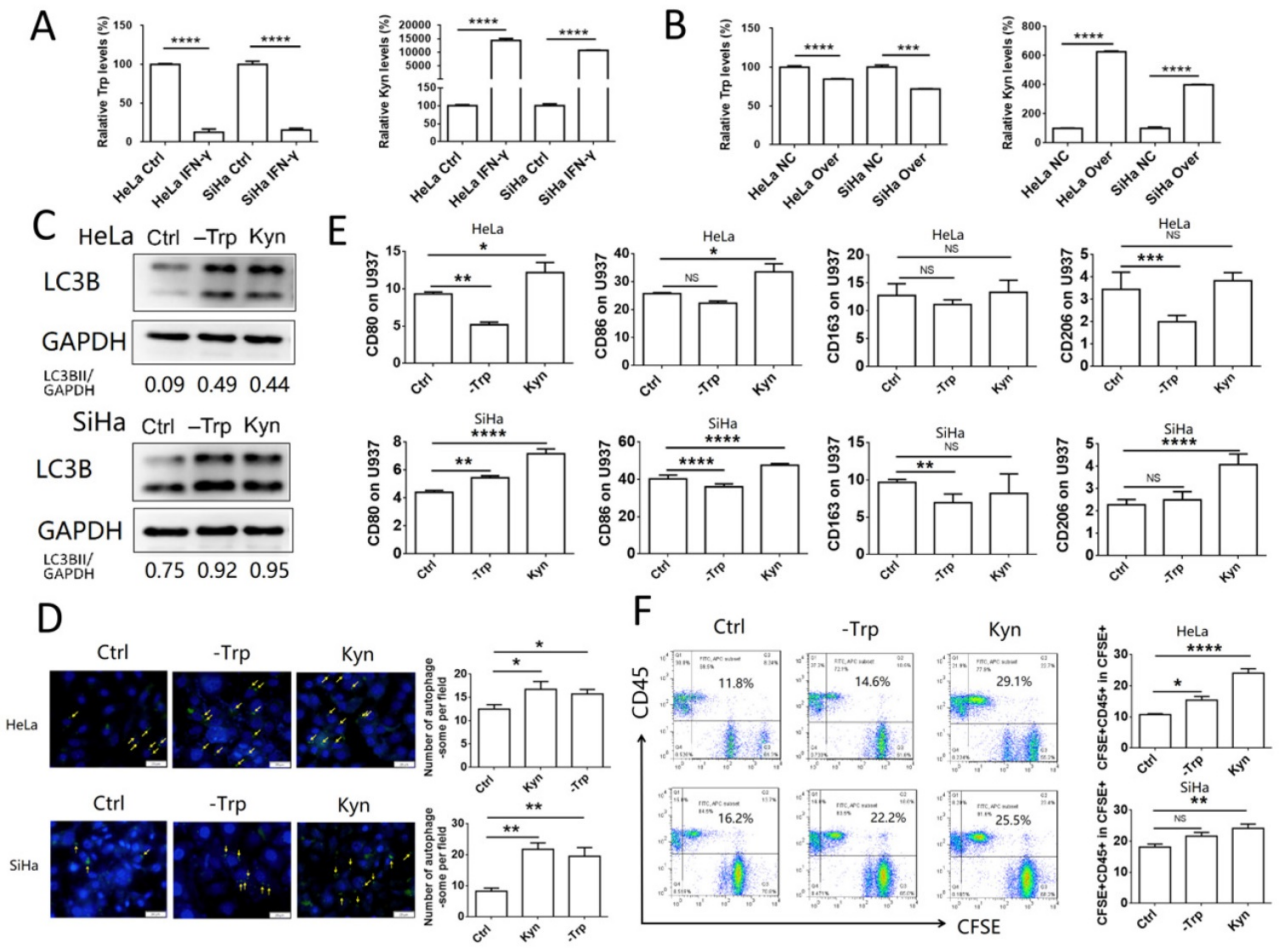
** The accumulation of kynurenine and consumption of tryptophan catalyzed by IDO1 promote phagocytosis and activation of macrophage.** (A) HeLa and SiHa cells were treated with IFN-γ or not; tryptophan and kynurenine level were measured by LC-MS/MS quantification. (B) HeLa and SiHa cells were transfected with IDO1 overexpression or negative control lentivirus; tryptophan and kynurenine level were measured by LC-MS/MS quantification. (C) HeLa and SiHa cells were treated with kynurenine (500μmol/l) or tryptophan free medium, and LC3B expression was measured by western blot. (D) HeLa and SiHa cells were treated with kynurenine or tryptophan free medium, and the autophagy level was detected by DAP autophagy kit. Autophagosome was observed with fluorescence microscope. Yellow arrows represented autophagosomes. Multiple fields of view were randomly selected through fluorescence microscope observation, and then the number of autophagosomes was calculated. (E) HeLa and SiHa cells were treated with kynurenine or tryptophan free medium, then co-cultured with U937 cells for 48 hours, and the CD80, CD86, CD163 and CD206 expression of U937 cells were measured by FCM. (F) HeLa and SiHa cells were treated with kynurenine or tryptophan free medium, then labelled with CFSE, and further co-cultured with U937 cells for 2 hours. The phagocytosis was tested by FCM. The results were expressed as mean ± SEM. -Trp represents for tryptophan free, Kyn represents for kynurenine. NS; not significant difference, * means *P<*0.05, ** means *P*<0.01, and **** means *P<*0.0001.

IDO1 is an enzyme which can catalyze the conversion of tryptophan to kynurenine. To assess the concentration levels of tryptophan and kynurenine in IFN-γ-treated or IDO1-overexpressed cervical cancer cells, mass spectrometric detection was used in samples isolated from HeLa and SiHa cells. The concentration of tryptophan was decreased significantly, whereas that of kynurenine was notably increased in IFN-γ-treated cancer cells, as well as in IDO1-overexpressing cells (**Fig. 5A-B**). These results were consistent with the data showing that IFN-γ treatment and IDO1 overexpression could increase IDO1 expression in HeLa and SiHa cells. Autophagy was detected in HeLa and SiHa cells treated with kynurenine (500 μmol/l) or tryptophan-free medium. The results indicated that both tryptophan depletion and kynurenine addition promoted the level of autophagy in cervical cancer cells (**Fig. 5C-D**). In addition, HeLa and SiHa cells pretreated with kynurenine exhibited a significant increase in CD80 and CD86 expression in U937 cells following 48 h of co-culture (**Fig. 5E**). The data further indicated that the effects of tryptophan depletion were not as obvious as those of kynurenine addition. The ratio of phagocytic cervical cancer cells in the kynurenine treatment group was increased significantly (**Fig. 5F**). Tryptophan depletion caused a significant upregulation of the autophagic markers in HeLa cells. This effect was considerably weaker following kynurenine addition (**Fig. 5F**). In conclusion, the data indicated that IFN-γ and IDO1 may promote the phagocytosis and activation of macrophages via the accumulation of kynurenine, rather than by the consumption of tryptophan.

### IDO1 inhibits tumor growth and promotes macrophage phagocytosis in vivo

**Figure 6:**
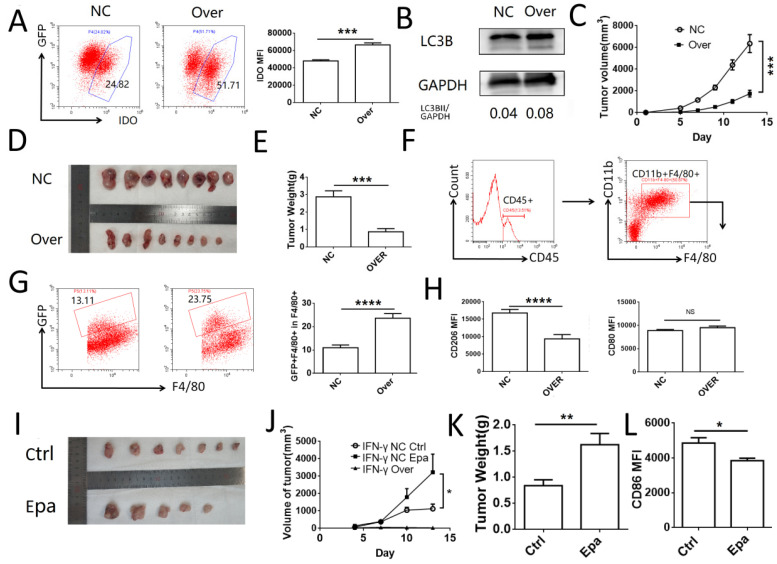
** IDO1 inhibits tumor growth and promotes macrophage phagocytosis *in vivo.*** (A) TC-1 cells were transfected with mus IDO1 overexpression or negative control lentivirus. And the IDO1 expression was measured by FCM. (B) LC3B in TC-1 cells transfected with lentivirus was measured by western blot. (C-E) TC-1 cells transfected with IDO1 overexpression or negative control lentivirus were injected subcutaneously in mice. Tumor volume was detected every other day. (F) Strategy of gating macrophage in tumor tissue. (G) Phagocytosis of macrophage towards GFP^+^ tumor cells were analyzed by FCM. (H) CD80 and CD206 were detected in macrophage (I-K) TC-1 cells transfected with IFNG overexpression or negative control lentivirus were injected subcutaneously in mice. And mice were administrated with epacadostat or control solvent every day. Tumor volume was detected every three days. (L) CD86 expression was detected in macrophage. The results were expressed as mean ± SEM. Epa represents for epacadostat. NS: not significant difference, * means *P<*0.05, ** means *P*<0.01, and **** means *P<*0.0001.

To investigate the role of IDO1 in cervical cancer cells *in vivo*, a subcutaneous tumor model was established in C57BL/6J mice using TC-1 cells transfected with IDO1 overexpressing or negative control lentiviruses. The efficiency of transfection was verified by FCM (**Fig. 6A**). The levels of TC-1 cell autophagy were detected by western blot analysis and the results were similar to those noted in HeLa and SiHa cells (**Fig. 6B**). The tumor volume and weight were significantly lower in the IDO1-overexpressing group compared to those noted in the control group (**Fig. 6C-E**). The detection of CD45^+^CD11b^+^F4/80^+^ cells was used to identify the macrophages in the tumor tissue. GFP^+^ macrophages were regarded as those, which had engulfed tumor cells (**Fig. 6F-G**). The number of TC-1 cells phagocytosed by macrophages in the IDO1-overexpressed group was elevated compared with that of the negative control group (**Fig. 6G**). Moreover, CD206 expression in macrophages of the IDO1-overexpression group was decreased, whereas CD80 expression was not significantly altered (**Fig. 6H**). Intraperitoneal injection of epacadostat caused a significant increase in tumor volume, tumor weight, while it concomitantly inhibited CD80 expression of macrophages in tumor tissues (**Fig. 6I-L**). However, intraperitoneal injection of IFN-γ or kynurenine did not reduce the growth of subcutaneous tumors in mice (**Fig. SF1**). This result may be associated with the route of administration and the direct effects of IFN-γ and kynurenine on immune cells.

### IFNG and IDO1 expressions are associated with a better survival in cervical cancer patients

**Figure 7:**
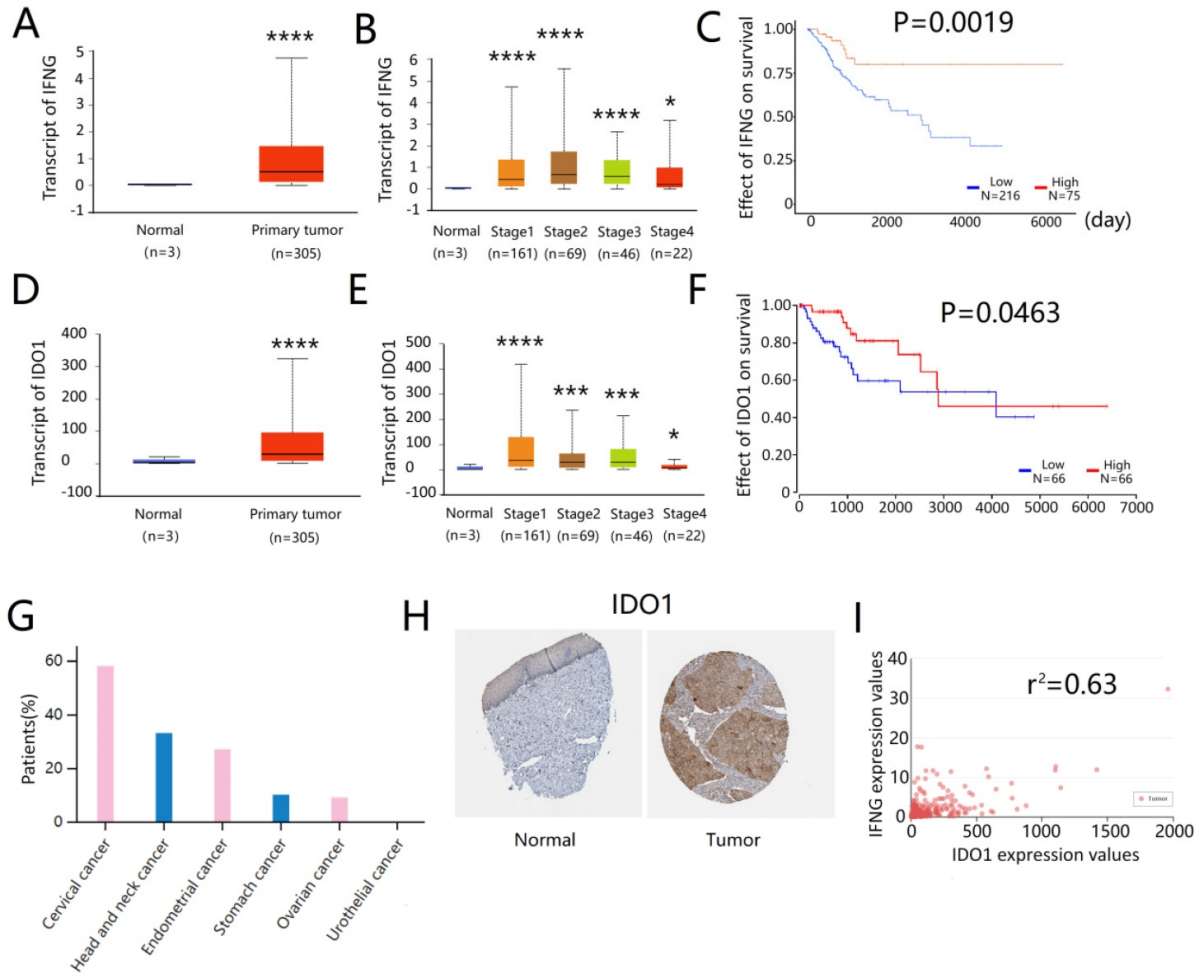
** IFNG and IDO1 expression were related to a better survival in cervical patients.** (A) IFNG expression in normal cervix and primary cervical cancer, data from TCGA database (http://ualcan.path.uab). (B) IFNG expression in normal cervix and primary cervical cancer based on the cancer stage (C) Kaplan plot of IFNG in cervical cancer (D) IDO1 expression in normal cervix and primary cervical cancer (E) IDO1 expression in normal cervix and primary cervical cancer based on the cancer stage (F) Kaplan plot of IDO1 in cervical cancer (G) ratio of IDO1 positive patients in cervical cancer, data from The Human Protein Atlas online database (https://www.proteinatlas.org/) (H) IDO1 expression in normal cervical epithelium and cervical cancer tissue. Tissue microarray was from The Human Protein Atlas (I) The correlation analysis of IFNG and IDO1 in cervical cancer. Data from TCGA database (http://ualcan.path.uab). * means *P<*0.05, and **** means *P<*0.0001.

The association of IFNG and IDO1 expressions on the survival of cervical cancer patients was assessed by the TCGA online database (http://ualcan.path.uab.edu). The expression levels of IFNG were significantly higher in the primary tumor than in the normal cervix samples (**Fig. 7A**). IFNG expression was also increased in all stages of cervical cancer (**Fig. 7B**). Higher IFNG expression was associated with a better patient survival (**Fig. 7C**). Similarly, IDO1 expression was also notably increased in cervical tumors than in normal cervix tissues (**Fig. 7D**). This effect was noted in all stages of tumors (**Fig. 7E**) and IDO1 expression was associated with a better survival (**Fig. 7F**). Immunohistochemical analysis of tissue arrays derived from HumanProteinAtlas (https://www.proteinatlas.org) indicated that more than half of the cervical cancer patients (7/12) expressed medium or high levels of IDO1 in tumor cells (**Fig. 7G**), whereas IDO1 protein expression levels were generally higher in cervical cancer tissues compared to those of the normal cervix tissues (**Fig. 7H**). Correlation analysis indicated that IDO1 expression in cervical cancer was associated with IFNG expression and the correlation coefficient was estimated to 0.63 (**Fig. 7I**). These results indicated that IDO1 expression in cervical cancer cells was associated with IFNG expression, whereas both IFNG and IDO1 expressions were associated with better survival of cervical cancer patients.

## Discussion

In the present study, the data demonstrated that HeLa and SiHa cells rarely expressed IDO1 without the stimulation of IFN-γ. IDO1 expression could be detected in cervical cancer tissues and was higher than that of the normal cervical epithelium. In addition, IDO1 expression was associated with a higher patient survival, notably in the first 3,000 days. A similar result was also reported by Venancio *et al*^17^. It has been shown that the expression of IDO1 in cervical tumor cells may be induced by the local microenvironment, such as IFN-γ and HPV 8, 18.

IDO1 has been shown to induce immune suppression in the local tumor environment by promoting the differentiation of regulatory T cells and by inhibiting the function of effector T cells 19. IDO1 is commonly co-expressed with programmed death ligand 1 (PD-L1) in cervical squamous carcinomas 20. However, in clinical studies, the combined treatment of patients with IDO1 and PD-L1 inhibitors did not significantly improve the outcome of the disease, whereas single treatment with the IDO1 inhibitor demonstrated minimal effects compared to those noted in the placebo group, in various tumor types including cervical cancer^21^. IDO1 is an enzyme that exists in almost all of the cells and its inhibition influences the metabolism of tryptophan in immune and tumor cells. As mentioned above, IDO1 induced of autophagy in cervical cancer cells, which further promoted phagocytosis and activation of macrophages in tumor tissues. Inhibition of IDO1 may impair the induction of autophagy in tumor cells, the phagocytotic activity of macrophages and even the antigen presenting function. Heeren *et al* demonstrated that IDO1^+^ tumors exhibited higher number of CD8^+^Ki67^+^ T cells (P=0.004) and that IDO1 expression was associated with improved disease-free (DFS) (P=0.017) and disease-specific survival (P=0.043) 8. This may partly explain why IDO1 inhibition could not increase the number of cervical cancer patients who achieved satisfactory treatment response. These results demonstrated the side effects of immunotherapy.

Autophagy is a conservative process, which occurs in eukaryocytes. The autophagosome contains cellular components that are delivered to lysosomes for degradation. This leads to basal recycling of various cellular parts, which in turn provides energy. It is universally accepted that autophagy actively exists in tumor cells. However, it is uncertain whether it participates in tumor progression 22. Previous studies have shown that autophagy in cancer inhibits the immune response, promotes tumor growth or induces drug resistance 23-26. It has also been shown that autophagy plays a role in stimulating tumor antigen cross-presentation, increasing immune cell activation and improving patient survival 15, 27, 28. Therefore, the role of autophagy is altered according to different environmental conditions. In the present study, the data demonstrated that in cervical cancer, autophagy promoted the phagocytosis of macrophages, induced the expression of CD80 and CD86 and resulted in antigen presentation and immune activation, which in turn limited the tumor growth.

IDO1 has been reported to induce autophagy by different mechanisms of action. The first one involves tryptophan depletion. Tryptophan is an essential amino acid in the human body. Its deficiency can cause the accumulation of non-charged Trp-tRNA in the cells, which is sensed by integrated stress response kinase General Control Non-depressible 2 (GCN2) 29. GCN2 is a potent driver of autophagy, which can initiate this process following its activation 30. Tryptophan-deficiency signaling causes autophagy via GCN2. This finding has been noted in T cells, podocytes and kidney epithelial cells. IDO1-GCN2-autophagy signals have been regarded as a common circuit in human inflammatory diseases 29, 31, 32. In addition, tryptophan deficiency signaling caused by IDO1 activity can lead to the inhibition of the activity of the mammalian target of rapamycin (mTOR) and protein kinase C independent of the GCN2 pathway 33. Inhibition of mTOR signaling was associated with decreased glycolysis, lower oxidative stress and increased autophagy 34.

The present study demonstrated that kynurenine accumulation was also an important signal disseminated in cells following IDO1 overexpression. Kondrikov *et al* demonstrated that at physiological levels (10 and 100 μmol/l), kynurenine could inhibit autophagy of bone marrow mesenchymal stem cells via Aryl hydrocarbon receptor (AhR) signaling 35. This study verified that the regulatory effect of kynurenine on autophagy was mediated via the AhR pathway. The present study indicated that in the presence of IFN-γ or following overexpression of IDO1, kynurenine concentration was increased to 100-fold or higher compared with the physiological concentration. The effects on autophagy may also differ under physiological and pathological conditions. Kynurenine is converted to specific metabolites, including kynurenic acid, quinolinic acid and NAD^+^. It was reported that kynurenine metabolites were associated with mitochondrial dysfunction and reactive oxygen species production, which may lead to cell autophagy 36. In the present study, kynurenine accumulation was more effective than tryptophan depletion. This result may be associated with the sensitivity of cervical cancer cells to the stimulation effect. Kynurenine-AhR signaling or the production of kynurenine metabolites in HeLa and SiHa cells may be more important in regulating the function of macrophages.

The induction of autophagy in tumor cells was involved in the antigen presentation. Li *et al* demonstrated that inhibition of autophagy in tumor cells abolished the cross-presentation caused by dendritic cells, whereas induction of autophagy enhanced the cross-presentation of tumor antigens. In addition, purified autophagosomes were found to be efficient antigen carriers for antigen presentation 37. Hahn *et al* demonstrated that alpha-tocopheryloxyacetic acid could promote antigen cross-presentation by triggering tumor autophagy. The autophagosome-enriched fractions of the tumor cells efficiently cross-primed antigen-specific CD8^+^T cells^38^. Similar results were also noted by Li *et al*^39^. These results demonstrated that autophagy in tumor cells promoted the antigen processing of antigen presenting cells. Phagocytosis of tumor cells and the expression of costimulatory molecules are of considerable importance in the process of antigen processing and presentation. Therefore, it is conceivable that autophagy can promote the phagocytosis and activation of macrophages. The mechanism of tumor cell-induced autophagy may regulate the function of macrophages. However, further studies are required to confirm this hypothesis.

IFN-γ and kynurenine effectively induced autophagy of cervical cancer cells *in vitro,* while they did not restrict tumor growth *in vivo*. Several reasons may contribute to this result. Intraperitoneal injection of IFN-γ may not be an ideal method of administration. This cytokine may be degraded inside the abdomen. Knockdown of IFN-γ or IFN receptor expression was more commonly used in previous animal experiments. An IFN-γ overexpressing TC-1 cell line was used and the data demonstrated that the tumor was not formed in the overexpression cell line model, whereas it did grow in the negative control cells. This may be partly attributed to the effect of IFN-γ. Intraperitoneal injection of kynurenine may influence both tumor cells and immune cells. Kynurenine may suppress the immune response by affecting the immune cells. In addition, the absorption of kynurenine by tumor cells may restrict its intracellular retention and low concentrations of kynurenine may not effectively induce autophagy in tumor cells.

The IDO1 inhibitor is not an ideal treatment for cervical cancer, whereas immunotherapy is still a significant treatment strategy used to improve the outcome of advanced cervical cancer patients. Bevacizumab and pembrolizumab are two approved therapies that have been added to the standard of care for these patients. Other drugs are currently under preliminary evaluation or undergoing clinical trials. An in-depth understanding of the molecular biology and relative biomarkers may aid the exploration of potential targets for immunotherapy. Effective activation of macrophages or inhibition of autophagy in tumor cells may be the key target of immunotherapy, which may offer treatment options in cervical cancer.

**Figure 8:**
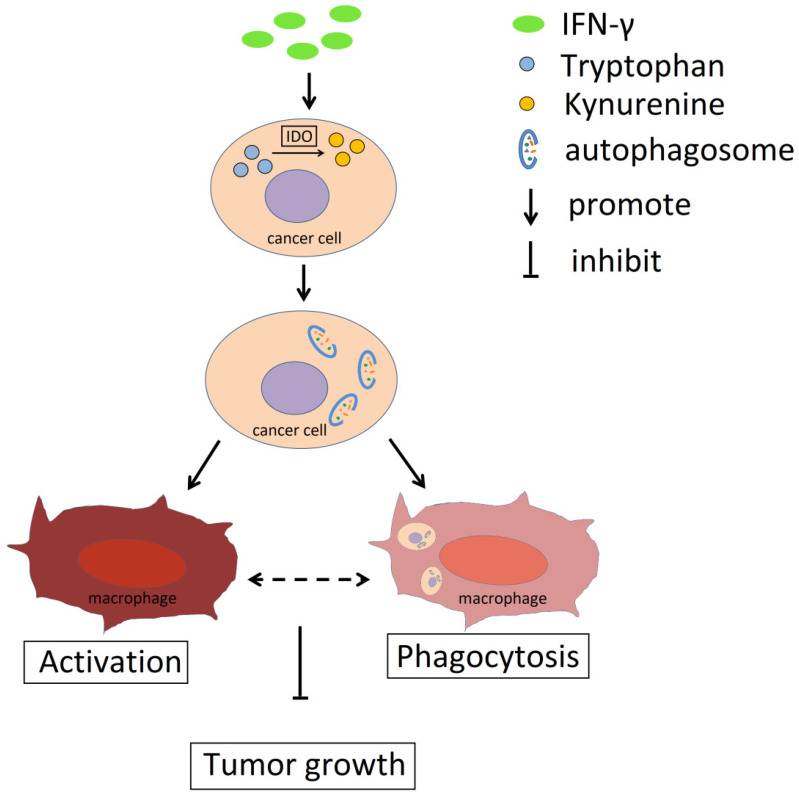
** Schema diagram of the effect of IFN**-**γ and IDO1 in cervical cancer.** IFN-γ promotes the IDO1 expression in cervical cancer cells, which could catalyze tryptophan into kynurenine. The accumulation of kynurenine and assumption of tryptophan in cancer cells induce the autophagy activity. The phagocytosis ability of macrophages towards tumor cells with active autophagy is stronger, and autophagy in cervical cancer cells promotes the activation of macrophage, which could be related to the phagocytosis towards cancer cells. As a result, IFN-γ could restrict the tumor growth through IDO1-kynurenine-autophagy pathway.

Collectively, the data indicated that IFN-γ could promote IDO1 expression in cervical cancer cells and induce its activity, which resulted in the conversion of tryptophan to kynurenine (**Fig. 8**). Accumulation of kynurenine promoted induction of autophagy in cervical cancer cells and further induced the activation and phagocytosis by macrophages. As a result, tumor growth was restricted. The activation and the phagocytosis of macrophages may interact with each other. Therefore, the IFN-γ-IDO1 axis-mediated kynurenine accumulation and enhanced the phagocytosis caused by macrophages, which in turn led to the inhibition of the progression of cervical cancer possibly by upregulating the levels of autophagy in cervical cancer cells.
